# A Retrospective Controlled Cohort Study of the Impact of Glucocorticoid Treatment in SARS-CoV-2 Infection Mortality

**DOI:** 10.1128/AAC.01168-20

**Published:** 2020-08-20

**Authors:** Ana Fernández-Cruz, Belén Ruiz-Antorán, Ana Muñoz-Gómez, Aránzazu Sancho-López, Patricia Mills-Sánchez, Gustavo Adolfo Centeno-Soto, Silvia Blanco-Alonso, Laura Javaloyes-Garachana, Amy Galán-Gómez, Ángela Valencia-Alijo, Javier Gómez-Irusta, Concepción Payares-Herrera, Ignacio Morrás-Torre, Enrique Sánchez-Chica, Laura Delgado-Téllez-de-Cepeda, Alejandro Callejas-Díaz, Antonio Ramos-Martínez, Elena Múñez-Rubio, Cristina Avendaño-Solá

**Affiliations:** a Infectious Diseases Unit, Internal Medicine Department, Hospital Universitario Puerta de Hierro-Majadahonda, Instituto de Investigación Sanitaria Puerta de Hierro-Segovia de Arana, Madrid, Spain; b Clinical Pharmacology Department, Hospital Universitario Puerta de Hierro-Majadahonda, Instituto de Investigación Sanitaria Puerta de Hierro-Segovia de Arana, Madrid, Spain; c Internal Medicine Department, Hospital Universitario Puerta de Hierro-Majadahonda, Instituto de Investigación Sanitaria Puerta de Hierro-Segovia de Arana, Madrid, Spain; d Pharmacy Department, Hospital Universitario Puerta de Hierro-Majadahonda, Instituto de Investigación Sanitaria Puerta de Hierro-Segovia de Arana, Madrid, Spain; e Medicine Department, School of Medicine, Universidad Autónoma de Madrid, Madrid, Spain

**Keywords:** COVID-19, steroids, mortality

## Abstract

Evidence to support the use of steroids in coronavirus disease 2019 (COVID-19) pneumonia is lacking. We aim to determine the impact of steroid use for COVID-19 pneumonia on hospital mortality. We performed a single-center retrospective cohort study in a university hospital in Madrid, Spain, during March of 2020. To determine the role of steroids in in-hospital mortality, patients admitted with severe acute respiratory syndrome coronavirus 2 (SARS-CoV-2) pneumonia and treated with steroids were compared to patients not treated with steroids, and we adjusted with a propensity score for patients on steroid treatment. Survival times were compared using the log rank test. Different steroid regimens were compared and adjusted with a second propensity score. During the study period, 463 out of 848 hospitalized patients with COVID-19 pneumonia fulfilled inclusion criteria. Among them, 396 (46.7%) patients were treated with steroids and 67 patients were not. Global mortality was 15.1%. The median time to steroid treatment from symptom onset was 10 days (interquartile range [IQR], 8 to 13 days). In-hospital mortality was lower in patients treated with steroids than in controls (13.9% [55/396] versus 23.9% [16/67]; hazard ratio [HR], 0.51 [95% confidence interval, 0.27 to 0.96]; *P* = 0.044). Steroid treatment reduced mortality by 41.8% relative to the mortality with no steroid treatment (relative risk reduction, 0.42 [95% confidence interval, 0.048 to 0.65]). Initial treatment with 1 mg/kg of body weight/day of methylprednisolone versus steroid pulses was not associated with in-hospital mortality (13.5% [42/310] versus 15.1% [13/86]; odds ratio [OR], 0.880 [95% confidence interval, 0.449 to 1.726]; *P* = 0.710). Our results show that the survival of patients with SARS-CoV-2 pneumonia is higher in patients treated with glucocorticoids than in those not treated. Rates of in-hospital mortality were not different between initial regimens of 1 mg/kg/day of methylprednisolone and glucocorticoid pulses.

## Abstract (precis)

Evidence to support the use of steroids in coronavirus disease 2019 (COVID-19) pneumonia is lacking. We aim to determine the impact of steroid use for COVID-19 pneumonia on hospital mortality. We performed a single-center retrospective cohort study in a university hospital in Madrid, Spain, during March of 2020. To determine the role of steroids in in-hospital mortality, patients admitted with severe acute respiratory syndrome coronavirus 2 (SARS-CoV-2) pneumonia and treated with steroids were compared to patients not treated with steroids, and we adjusted with a propensity score for patients on steroid treatment.

## INTRODUCTION

Infection with severe acute respiratory syndrome coronavirus 2 (SARS-CoV-2) presents mainly with respiratory involvement. Clinical presentation consists of a first viremic phase that is 7 to 10 days long, followed in some cases by a second phase of clinical manifestations driven by lung and systemic inflammation (1). During the initial viremic phase, antiviral drugs are recommended, especially in cases with pneumonia. Around 80% of cases will resolve after this first phase. However, another 20% will evolve to a severe pneumonitis, followed by acute respiratory distress syndrome (ARDS). In that second phase, an increase in acute-phase reactants and macrophage activation markers has been identified. Poor outcomes have been associated with high interleukin 6 (IL-6) levels, leading to the recommendation of treatment with IL-6 antagonists.

The scarcity of anti-inflammatory targeted therapies, such as tocilizumab, during the initial period of the SARS-CoV-2 pandemic has driven the use of glucocorticoids in these patients, particularly in the more severe cases as a last resort, despite the recommendation against it. Based on studies performed during the prior SARS-CoV, Middle East respiratory syndrome CoV (MERS-CoV), and H1N1 influenza epidemics, the WHO advised against use of glucocorticoids in COVID-19 patients owing to a possible deleterious effect of the prolongation of viral excretion and increased adverse events (2). The available studies have important methodologic limitations, and of note, glucocorticosteroids (hereinafter “steroids”) were usually administered early after symptom onset (4 days) (3). Nevertheless, in the current pandemic, the Chinese National Commission recommended methylprednisolone at 1 to 2 mg/kg of body weight/day for 3 to 5 days in cases with respiratory failure (4), and several studies suggest a possible beneficial effect of steroids administered in the inflammatory phase of the disease in patients with ARDS (5, 6). In this respect, the use of steroids as an adjuvant therapy in cases of moderate to severe ARDS is accepted in the early stages at a dosage of 1 mg/kg/day of methylprednisolone in intubated patients (7). In severe and rapidly progressive ARDS, methylprednisolone appears to improve symptoms and pulmonary damage but does not increase survival (8). Nevertheless, this inhibition of the inflammatory storm may allow us to gain time to control the infection and prevent secondary multiorgan failure and shock (9). Recently, early administration of dexamethasone has shown a survival advantage in cases of established moderate to severe ARDS (10). Moreover, the combined effect of steroids with other anti-inflammatory therapies used concomitantly, namely, in those cases in which the targeted therapy needs some time to achieve a response, is still to be determined (9).

However, there is a lack of evidence to support steroid use, and there also is uncertainty about the most appropriate drug, dose, and timing. It is unknown whether the appropriate steroid dose might be the same in different stages of the disease and what the therapeutic ceiling is. While awaiting results from ongoing clinical trials (11), we consider that an analysis of actual clinical practice is needed to guide the recommendations. We performed a retrospective analysis of our experience to test the hypothesis that steroid use can improve the mortality of patients with COVID-19 pneumonia.

## RESULTS

During the study period, 848 patients with COVID-19 and pneumonia were admitted to the hospital. Four hundred sixty-three out of 848 patients (55%) were included. Among them, 396 were treated with steroids, while 67 were assigned to the control cohort. A total of 385 patients that were excluded from participation were hospitalized with COVID-19 but did not develop ARDS or exhibit increases in inflammatory markers.

### Clinical characteristics.

Clinical characteristics of the cases and controls are displayed in Table 1. The median time to steroid treatment from the onset of symptoms was 10 days (interquartile range [IQR], 8 to 13 days). Among patients treated with steroids, 310 (78.3%) patients were initially treated with 1 mg/kg/day methylprednisolone or the equivalent (22.5% of them received steroid pulses later on) and 86 (21.7%) received pulses from the beginning.

**TABLE 1:** Baseline demographic and clinical characteristics of the patients in both cohorts^*a*^

Parameter (463 patients)	Steroid cohort (*n* = 396)	Control cohort (*n* = 67)	*P* value
No. (%) of male patients	276 (69.7)	41 (61.2)	0.200
Mean age (yr) (SD)	65.4 (12.9)	68.1 (15.7)	0.132
Mean Charlson score (yr) (SD)	2.0 (2.3)	2.3 (2.6)	0.389

No. (%) of patients with underlying medical conditions	306 (77.3)	53 (79.1)	0.874
High blood pressure	182 (46.0)	32 (47.8)	0.793
Ischemic heart disease	72 (18.2)	12 (17.9)	0.957
Diabetes	84 (21.2)	13 (19.4)	0.871
Obesity	29 (7.3)	6 (9.0)	0.619
Dyslipidemia	113 (28.5)	22 (32.8)	0.471
Cardiovascular risk factors	249 (63.2)	48 (71.6)	0.215
Chronic kidney disease	24 (6.1)	4 (6.0)	0.977
Onco-hematologic disease	49 (12.4)	16 (23.9)	***0.021***
COPD	71 (17.9)	10 (14.9)	0.607
Transplant (SOT/SCT)	9 (2.3)	1 (1.5)	0.685
Neurologic disease	35 (8.8)	11 (16.4)	0.074
Rheumatologic disease	14 (3.5)	1 (1.5)	0.707
Hepatic disease	10 (2.5)	5 (7.5)	0.051
Peptic ulcer disease	3 (0.8)	3 (4.5)	***0.013***
Thromboembolic disease	5 (1.3)	3 (4.5)	0.062
Thyroid disorders	15 (3.8)	5 (7.5)	0.189
Immunosuppression	37 (9.4)	4 (6.0)	0.171

No. (%) with indicated clinical symptom at admission:			
Cough	312 (79.6)	44 (65.7)	***0.017***
Fever	353 (90.3)	56 (83.6)	0.131
Dyspnea	272 (69.2)	42 (62.7)	0.321
Gastrointestinal problem	92 (23.2)	13 (19.4)	0.532
Sore throat	22 (5.6)	4 (6.0)	0.780
Anosmia/ageusia	28 (7.1)	5 (7.5)	0.802
Myalgia	82 (20.7)	12 (17.9)	0.743
Headache	29 (7.3)	4 (6.0)	0.691
Fatigue	63 (15.9)	15 (22.4)	0.216
Chest pain	23 (5.8)	3 (4.5)	0.662
Rash	2 (0.5)	0	0.560
Increased sputum production	6 (1.5)	5 (7.5)	***0.013***
Confusion	18 (4.7)	10 (15.4)	***0.003***

Mean no. of days (SD) from onset of symptoms to:			
Diagnosis	8.5 (5.1)	6.9 (3.9)	***0.021***
Hospital admission	7.6 (4.2)	7.0 (3.7)	0.231
Therapy	7.4 (4.1)	7.1 (3.6)	0.506
Inclusion	10.8 (4.8)	8.7 (4.4)	***0.002***

No. (%) of patients treated with:			
Hydroxychloroquine	393 (99.5)	62 (92.5)	***0.001***
Lopinavir-ritonavir	287 (73.0)	42 (62.7)	0.106
Azithromycin	208 (53.9)	29 (43.9)	0.144
Interferon	186 (47.6)	28 (41.8)	0.427
Tocilizumab	177 (44.9)	12 (18,5)	***<0.001***
Anakinra	8 (2.0)	0	0.241
Other treatments^*b*^	65 (16.4)	20 (29.9)	***0.009***
			
Mean PaO_2_/FiO_2_ (SD)	263 (112.1)	267 (78.9)	0.878
Mean SatO_2_/FiO_2_ (SD)	286 (123.0)	244 (91.9)	***0.021***
Mean no. (%) of patients with a Brescia-COVID-19 score of >2	77 (17.4)	16 (23.9)	0.411

No. (%) of patients with ARDS			***<0.001***
No	156 (39.4)	9 (13.4)	
Mild	96 (24.2)	43 (64.2)	
Moderate	116 (29.3)	15 (22.4)	
Severe	28 (7.1)	0	
Admitted to ICU at day 0	30 (7.6%)	0	***0.013***
Mean result (SD) from laboratory test (day 0) for:			
Lymphocyte counts	1,004 (1,354)	1,190 (1,042)	0.342
Lactate dehydrogenase	396 (154)	338 (117)	***0.018***
d-Dimer	2.5 (7.6)	2.1 (4.6)	0.741
C-reactive protein	141 (85)	122 (76)	0.157
Ferritin	1,353 (2.220)	763 (1.008)	0.347
IL-6	196 (228)	62 (62)	***0.039***

No. (%) of patients with indicated chest CT result (at hospital admission)			***0.033***
Normal	16 (4.1)	2 (3.1)	
Unilateral pneumonia	29 (7.5)	12 (18.5)	
Bilateral interstitial pneumonia	217 (55.8)	27 (41.5)	
Patchy bilateral pneumonia	93 (23.9)	16 (24.6)	
Confluent bilateral pneumonia	34 (8.7)	8 (12.3)	

a COPD, chronic obstructive pulmonary disease; SOT, solid organ transplantation; SCT, stem cell transplantation; D0, day 0; PaO_2_/FiO_2_, arterial oxygen tension/inspiratory oxygen fraction; SatO_2_/FiO_2_, oxygen saturation/inspiratory oxygen fraction; ARDS, acute respiratory distress syndrome; ICU, intensive care unit; Brescia-COVID-19, Brescia-COVID-19 respiratory severity scale; IL-6, interleukin 6; CT, computed tomography scan. Boldface italics indicate significant *P* values.

b Including ritonavir-boosted darunavir, doxycycline, or clarithromycin and other antibiotics.

Patients treated with steroid pulses received a median of 3 pulses (IQR, 2 to 4 pulses), followed by tapering in 25% of cases. Pulses of methylprednisolone were classified in the following groups: <250 mg/day (20.1%), 250 mg/day (62.5%), and 500 mg/day (17.1%).

Forty-one patients (8.9%) in our series, including patients that had received prior steroid treatment, were considered immunosuppressed. Of these, 10 (24.4%) had underlying rheumatologic conditions, 16 (39.0%) had underlying onco-hematologic conditions, and the remaining patients presented other conditions that involved the administration of immunosuppressive treatment. The percentages of immunosuppressed patients in both cohorts were similar (9.3% of the steroids cohort versus 6.0% of the control cohort; *P* = 0.369).

### In-hospital mortality of patients treated with steroids compared to patients not treated with steroids.

The global in-hospital mortality was 15.3%. Characteristics of survivors and nonsurvivors are shown in Table S1 in the supplemental material.

**FIG 1:**
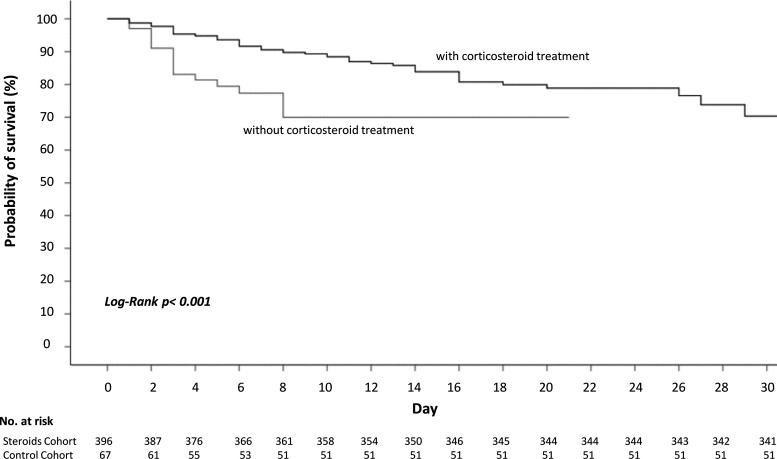
Probability of survival from D0 to hospital discharge of patients with SARS-COV-2 infection, according to steroid exposure.

In-hospital mortality was lower in patients treated with steroids than in controls (13.9% versus 23.9%; hazard ratio [HR], 0.51 [95% confidence interval, 0.27 to 0.96]; *P* = 0.044) (Table 2). Steroid treatment reduced mortality by 41.8% relative to no steroid treatment (relative risk reduction, 0.42 [95% confidence interval, 0.048 to 0.65]). We calculated a number necessary to treat 10. A propensity score to reduce the effect of steroid treatment selection bias was developed. Significant differences in baseline characteristics between steroid-treated and nontreated patients, such as onco-hematologic underlying conditions, peptic ulcer disease, lactate dehydrogenase (LDH), and oxygen saturation (SpO_2_), were considered for the propensity score. Peptic ulcer disease is considered a relative contraindication for steroid use, and this may have influenced an inferior steroid use in patients with a history of this condition. The difference in mortality persisted after applying the propensity score adjusted for steroid treatment (Table 2). Figure 1 demonstrates differences in the probabilities of survival at day 30 for patients with SARS-CoV-2 infection, according to steroid treatment (log rank *P* < 0.001).

**TABLE 2:** Association between steroid treatment and mortality in patients with SARS-COV-2 infection, according to steroid exposure and steroid regimen

Steroid exposure	No. (%) of survivors (*n* = 392)	No. (%) of nonsurvivors (*n* = 71)	HR (95% CI)	*P* value
No corticosteroid treatment	51 (76.1)	16 (23.9)	0.514 (0.274–0.965)	***0.038***^*a*^
Steroid treatment	341 (86.1)	55 (13.9)		

Steroid treatment (adjusted by PSM^*c*^)			0.360 (0.139–0.932)	***0.035***^*a*^

Steroid regimen				
1 mg/kg/day	268 (86.5)	42 (13.5)	0.880 (0.449–1.726)	0.71^*b*^
Pulses	73 (84.9)	13 (15.1)		

a Comparison of patients given and not given steroid treatment (any regimen). Boldface italics indicate significant *P* values.

b Comparison of the results from an initial 1 mg/kg/day and initial steroid pulses.

c PSM, propensity score matching.

**FIG 2:**
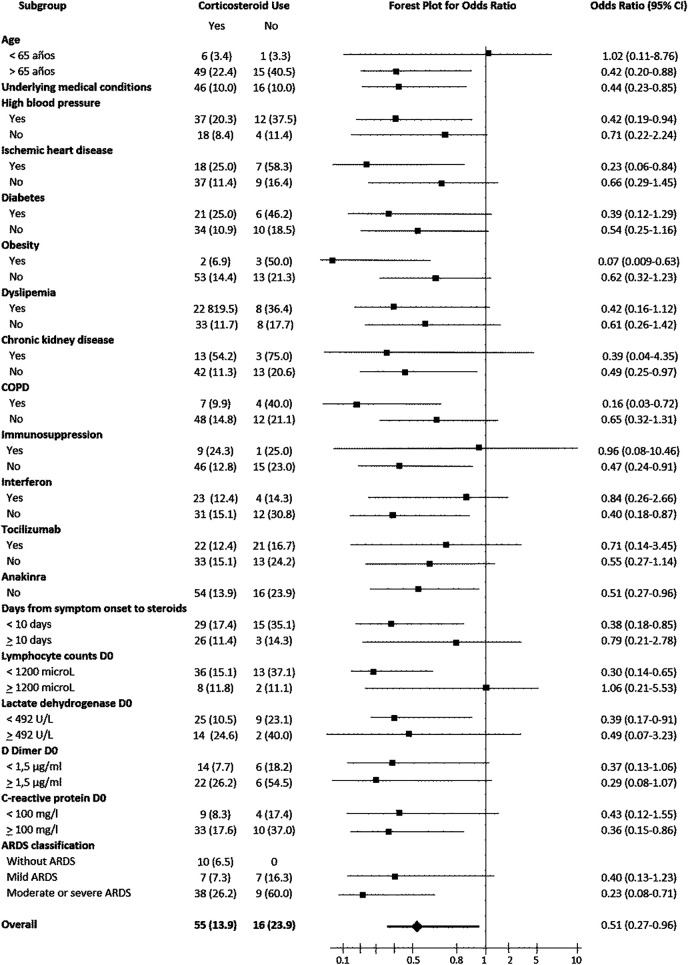
Forest plot of stratified analyses for in-hospital mortality showing the adjusted odds ratios of corticosteroid treatment. The subgroups were classified by demographic and disease characteristics.

Among patients with moderate or severe ARDS, in-hospital mortality was lower in patients treated with steroids than in the controls (26.2% versus 60%; odds ratio [OR], 0.23 [95% confidence interval, 0.08 to 0.71]; *P* = 0.014). Table S1 in the supplemental material and Fig. 1 and 2 show differences in probabilities of survival at day 30 according to steroid treatment, stratified according to ARDS severity.

The effect of steroid treatment on mortality in different subsets of patients was consistent with a protective effect (Fig. 2).

Table 3 shows the risk factors for mortality in both univariable and multivariable analyses, including those adjusted by the propensity score for steroid treatment. Older age, chronic kidney disease, more severe ARDS, and elevated LDH levels were independent risk factors for mortality, whereas steroid treatment was an independent protective factor. These results were confirmed when adjusted by propensity score for steroid treatment except for ARDS severity.

**TABLE 3:** Univariable and multivariable analyses of factors associated with hospital mortality in patients with SARS-COV-2 infection^*a*^

Variable	Univariable analysis		Multivariable analysis		Multivariable adjusted according to propensity score	
	OR (95% CI)	*P* value	OR (95% CI)	*P* value	OR (95% CI)	*P* value
Age	1.12 (1.08–1.15)	***<0.001***	1.13 (1.08–1.18)	***0.048***	1.11 (1.05–1.18)	***<0.001***
Age-adjusted Charlson score	1.34 (1.21–1.49)	***<0.001***				
Underlying medical conditions	3.6 (1.5–8.6)	***0.002***				
High blood pressure	3.1 (1.8–5.3)	***<0.001***				
Ischemic heart disease	3.1 (1.7–5.4)	***<0.001***				
Diabetes	2.8 (1.6–4.9)	***<0.001***				
Dyslipidemia	2.0 (1.2–3.4)	***0.011***				
Cardiovascular risk factors	2.5 (1.4–5.7)	***0.003***				
Chronic kidney disease	9.2 (4.1–20.5)	***<0.001***	29.13 (7.93–107.03)	***<0.001***	43.28 (6.90–273.87)	***<0.001***
Onco-hematologic disease	2.5 (1.3–4.6)	***0.005***				
Transplant (SOT/SCT)	8.9 (2.5–32.6)	***0.001***				
Neurologic disease	3.1 (1.6–6.1)	***0.002***				
Hydroxychloroquine	0.13 (0.28–0.59)	***0.013***				
Lopinavir-ritonavir	0.42 (0.25–0.70)	***0.001***				
PaO_2_/FiO_2_ (on D0)	0.99 (0.98–0.99)	***<0.001***				
SatO_2_/FiO_2_ (on D0)	0.99 (0.991–0.998)	***<0.001***				
ARDS	1.77 (1.01–3.09)	***0.046***	2.00 (1.14–3.51)	***0.015***	1.17 (0.51–2.67)	0.714
Lactate dehydrogenase (on D0)	1.003 (1.001–1.005)	***0.001***	1.004 (1.00–1.01)	***0.002***	1.001 (1.00–1.01)	***0.012***
d-Dimer (on D0)	1.04 (1.00–1.07)	***0.036***				
C-reactive protein (on D0)	1.004 (1.001–1.008)	***0.011***				
Steroid treatment	0.51 (0.27–0.96)	***0.044***	0.34 (0.12–0.99)	***0.048***	0.19 (0.05–0.74)	***0.016***

a SOT, solid organ transplantation; SCT, stem cell transplantation; D0, day 0; PaO_2_/FiO_2_, arterial oxygen tension/inspiratory oxygen fraction; SatO_2_/FiO_2_, oxygen saturation/inspiratory oxygen fraction; ARDS, acute respiratory distress syndrome.

### In-hospital mortality of patients treated with different steroid regimens: pulses versus 1 mg/kg/day.

Characteristics of patients initially treated with 1 mg/kg/day of methylprednisolone (or the equivalent) versus those initially treated with steroid pulses are displayed in Table S2. Being treated with either regimen was not associated with in-hospital mortality (13.5% versus 15.1%; OR, 0.880 [95% confidence interval, 0.449 to 1.726]; *P* = 0.710; RRR, 0.10 [95% confidence interval, –0.59 to 0.50]).

A propensity score for the choice of initial steroid regimen was developed. After adjusting for this propensity score, there were still no differences in mortality rates.

### Characteristics of patients initially treated with 1 mg/kg/day that eventually required salvage steroid pulses.

A subset of patients initially treated with 1 mg/kg/day of methylprednisolone received subsequent steroid pulses for a median time of 3 days (IQR, 2 to 7 days). Baseline characteristics of these patients, as opposed to those who did not, are presented in Table S3. Diabetic patients and those with underlying neurologic disease or higher levels of LDH at steroid initiation were more prone to require subsequent pulses, according to the multivariable analysis.

## DISCUSSION

Our results show that survival of patients with SARS-CoV-2 pneumonia is higher in patients treated with steroids than in those not treated. These results support the use of steroids in SARS-CoV-2 infection. Rates of in-hospital mortality were not different between patients treated with initial regimens of 1 mg/kg/day of methylprednisolone and steroid pulses.

The timing of the steroid administration might be decisive. In the present study, patients received steroid treatment for a median of 10 days after the onset of symptoms, presumably during the inflammatory phase of the disease. Three distinct stages of COVID-19 illness have been suggested (1). Siddiqi and Mehra (1) suggested that from stage IIB on, starting when hypoxia develops, anti-inflammatory therapies such as steroids may be beneficial due to the predominant role of inflammation in its pathophysiology.

The warning against the use of steroids in COVID-19 is based on studies that administered this therapy earlier during the course of the disease and relies on the experience from different viruses (12). Moreover, it has been speculated that steroid administration in patients with SARS-CoV-2 infection may be deleterious due to an increase of viral shedding or a delay in viral clearance. Although this theory was not confirmed in a recent work by Fang et al. (13), it is worth considering whether steroids are to be administered early on in the course of the disease. As referred to by Shang et al. (14), the evidence about the use of steroids is inconclusive and randomized controlled trials are needed.

In the present series, steroids were used in patients with hypoxemia that were not at an early phase of the disease, as stated by the median time from the onset of symptoms to steroid administration. As many as 64% of the cases fulfilled ARDS criteria at the time of steroid administration. At this stage, as suggested by the lower rates of mortality seen in the treatment cohort than in the control group, steroid treatment was beneficial. In general, guidelines recommend not using corticosteroids in patients with COVID-19 or using them only in intubated patients (15) or in the setting of randomized clinical trials (16). In our series, steroid treatment was beneficial in patients with moderate to severe ARDS, but a trend to a better survival was also seen in cases with mild ARDS, though it did not reach statistical significance, possibly due to a small sample size. When steroids are delayed to more advanced stages, we might miss a therapeutic window to prevent the evolution to severe ARDS and the need for mechanical ventilation. Nevertheless, the optimal stage for steroid treatment remains to be elucidated.

Patients with higher LDH levels responded better to steroid treatment in the present series. As LDH can be considered a surrogate marker for the extent of lung involvement, these results indicate that patients with more extensive lung damage might benefit more from steroid treatment. In this respect, our results are in line with those reported by others (5).

A pattern of cytokines resembling that of secondary hemophagocytic lymphohistiocytosis has been associated with SARS-CoV-2 infection (17). Mehta et al. (18) suggested a role for corticosteroids in patients with severe COVID-19 and hyperinflammation diagnosed based on their cytokine elevation profile. In our series, steroids’ protective effect was more intense in cases with higher d-dimer and C-reactive protein levels.

Optimal steroid dosing also needs clarification. Most patients treated with steroids in the present series received a weight-adjusted dose, but a significant proportion of patients (39.4%) received higher doses in pulses, either from the start of therapy or as salvage therapy, after a weight-adjusted course. In our series, we were not able to demonstrate a difference in mortality between these two regimens, even after adjusting by determining a propensity score taking into consideration the regimen choice and disease severity. An analysis of secondary adverse effects, which are usually dose related, would help to decide between regimens if both dosing regimens are confirmed to be associated with equivalent outcomes.

Our results are in line with a more preliminary work by Wang et al. (19), who reported a shorter duration of fever and a faster improvement of SpO_2_ in cases of severe SARS-CoV-2 pneumonia treated with 1 to 2 mg/kg/day of methylprednisolone during a period of 5 to 7 days. The present study has a considerably larger sample size, a more diverse population, and the added value of a propensity score to adjust for steroid treatment. Moreover, we report an impact on in-hospital mortality.

A study by Zhou et al. (9), which included only critical patients and lacked a control group, suggested that steroid treatment could enhance oxygen saturation and the partial pressure of arterial oxygen (PaO_2_)/percentage of inspired oxygen (FiO_2_) ratio, although mortality remained similar to that reported in the literature. Our study suggests that besides intensive care unit (ICU) patients with severe ARDS, other subsets of patients in an earlier phase of the disease may benefit from steroid therapy and possibly avoid ICU admission.

Other treatment-related factors, such as hydroxychloroquine or tocilizumab use, that might influence mortality and were not evenly distributed with regard to exposure to steroids, were not independent predictors for mortality in the multivariable analysis.

The present study is a retrospective study that analyzes real-life data, and as such, treated and untreated patients are not comparable according to all baseline characteristics. To overcome this limitation, we applied two propensity scores to the analysis, one for steroid treatment versus no steroid treatment and a second one for the initial steroid regimen choice. Results were confirmed when the propensity scores were included. As a single-center study, the results need external validation. The only outcome that was evaluated in the study was mortality. We consider that ICU admission during the study period is not a reliable marker of poor outcome, given the scarcity of available ICU beds during the critical moments of the pandemic, which forced us to apply strict restrictions for ICU admission.

The potential impact of steroids in the mortality of patients with COVID-19 pneumonia suggested by this study supports the need to carry out randomized clinical trials with the aim to establish their role. The optimal timing for administration, the subset of patients with the best risk/benefit ratio, and the appropriate dosing and duration remain to be elucidated.

## MATERIALS AND METHODS

### (i) Design, study period, and subjects.

This single-center retrospective cohort study included patients admitted to Hospital Puerta de Hierro-Majadahonda between 4 March 2020 and 7 April 2020. Our institution is a 613-bed tertiary-care teaching hospital in Madrid, Spain.

Adult patients diagnosed with COVID-19 pneumonia according to WHO interim guidance and complicated with ARDS and/or an hyperinflammatory syndrome where included. Of them, patients who received corticosteroid therapy according to clinical practice were assigned to the steroid cohort, while patients who did not were assigned to the control cohort.

### (ii) Data collection.

Epidemiological, clinical, laboratory, and radiologic data were extracted from electronic medical records (SELENE System, Cerner Iberia, S.L.U., Madrid, Spain) using a standardized data collection form. All data were included by a primary reviewer and subsequently checked by two senior physicians.

### (iii) Laboratory procedures.

Routine blood examinations included a complete blood count, a coagulation profile, serum biochemical tests (including for lactate dehydrogenase), and tests for C-reactive protein, d-dimer, interleukin-6 (IL-6), and serum ferritin. Chest radiographs or computed tomography (CT) scans were also done for all inpatients.

### (iv) Definition of the outcome.

The main outcome variable was in-hospital mortality. The outcomes of patients treated with steroids were compared to those of patients who did not receive steroids.

### (v) Definition of the exposure.

Exposure to corticosteroids was defined as the use of intravenous steroids at any time during the hospital admission.

Patients given steroid treatment were designated the treatment cohort, and those who did not receive steroids were designated the control cohort.

The decision to prescribe steroids was at the discretion of the treating physician, as the use of corticosteroids was not included in the COVID-19 local protocol at the time of the study. Details of corticosteroid use (including the timing of initiation, dosing, and type of medications) were recorded. Likewise, the choice of COVID-19 treatments other than corticosteroids was at the discretion of the treating physician although based on national and local recommendations for COVID-19 management. There were patients who had received prior steroid treatment due to chronic conditions (typically oral steroids). If steroid doses were modified with the aim of treating COVID-19, they were included as cases. If they continued with their usual steroid dose, they were included as controls.

In the treatment cohort, the first day of administration was considered the index date (day 0). In the control cohort, the index date was selected as the date at which the patient fulfilled ARDS criteria or presented any inflammation-related parameter level over the limits of the normal range. The diagnosis and grading of ARDS was determined according to modified Berlin criteria (20) (as most patients were not ventilated, the positive-end expiratory pressure [PEEP] value in the modified criteria was not taken into consideration).

For the main analysis, we generated a variable with the following mutually exclusive categories: nonuse of steroid drugs (control cohort) and use of steroid drugs (treatment cohort). Subsequently, we disaggregated the latter into two different subgroups: those given 1 mg/kg/day methylprednisolone or the equivalent and those given a steroid pulse. When a patient received different corticosteroid regimens during hospitalization, the first prescribed regimen was considered for the analysis.

### (vi) Statistical analysis.

Quantitative variables are expressed as means and standard deviations (SD) and/or medians and interquartile ranges, and qualitative variables are expressed as frequencies and percentages. The association of comorbidities among the treatment and control cohorts with mortality was assessed through univariable conditional logistic regression to compute crude odds ratios (ORs) and their 95% confidence intervals (95% CIs). Survival times were estimated using the Kaplan-Meier method, and differences between the cohorts were compared using a log rank test. The Mann-Whitney U test, χ^2^ test, or Fisher’s exact test was used to compare differences between survivors and nonsurvivors, where appropriate. To explore risk factors associated with in-hospital death, univariable and multivariable logistic regression models were used. Variables with a *P* of <0.05 in univariable models were selected into the multivariable analysis.

To reduce the effect of corticosteroid treatment selection bias and potential confounding, we adjusted for differences in baseline characteristics by a propensity score, which predicts the patient’s probability of being treated with steroids regardless of confounding factors, using multivariable logistic regression. Potential confounders considered in propensity score matching (PSM) analysis were those variables included in the final model by means of stepwise backward elimination procedures. The effect of corticosteroid treatment on clinical outcome was analyzed by multivariable logistic regression, adjusted for major variables associated with mortality; the individual propensity score was incorporated into the model as a covariate to calculate the propensity-adjusted OR.

Likewise, a second propensity score was developed to adjust for the choice of initial steroid regimen.

All statistical analyses were performed using an SPSS system (version 26.0 for Windows; SPSS Inc., Chicago, IL, USA). The statistical significance level was set at a two-sided *P* value of <0.05. A hazard ratio (HR) or an OR was reported along with the 95% CI.

### (vii) Ethics.

The study was approved by the Institutional Review Board (CEIm) at Hospital Universitario Puerta de Hierro-Majadahonda (BRA-COR-2020-03), and a waiver for informed consent was granted. The study complied with the provisions in European Union (EU) and Spanish legislation on data protection and the Declaration of Helsinki (21).

### (viii) Registration.

The protocol of the study was registered with EU Register of Post-Authorisation Studies (PAS Register) number EUPAS34753 on 15 April 2020 and is publicly available at http://www.encepp.eu/encepp/studySearch.htm.

### (ix) Data sharing.

After publication, the data will be made available to others upon reasonable requests to the corresponding author. A proposal with a detailed description of study objectives and statistical analysis plan will be needed for evaluation of the reasonability of requests. Additional materials might also be required during the process of evaluation. Deidentified participant data will be provided after approval from the principal researchers of Hospital Universitario Puerta de Hierro-Majadahonda.

## Supplementary Material

Supplemental file 1
