# Supplementary material for: A Retrospective Controlled Cohort Study of the Impact of Glucocorticoid Treatment in SARS-CoV-2 Infection Mortality
Source: Antimicrob Agents Chemother. 2020 Aug 20;64(9):e01168-20. doi: 10.1128/AAC.01168-20 (PMC7449182; doi:10.1128/AAC.01168-20)
Supplement: Supplemental file 1 [file AAC.01168-20-s0001.pdf]

Appendix Table 1. Comparison between survivors and non-survivors

N=463	Survivors (N =392)	Non-survivors (N = 71)	p value
Gender (Men), n (%)	270 (68.9)	47 (66.2)	0.678
Age, Mean (SD)	63.6 (12.6)	77.8 (10.4)	**< *0.001***
Charlson score, Mean (SD)	3..6 (2..8)	1.7 (2..1)	**< *0.001***
Underlying Medical Conditions, n (%)	294 (75.0)	65 (91.5)	***0.002***
High Blood pressure	165 (42.1)	49 (69.0)	**< *0.001***
Ischemic heart disease	59 (15.1)	25 (35.2)	**< *0.001***
Diabetes	70 (17.9)	27 (38.0)	**< *0.001***
Obesity	30 (7.7)	5 (7.0)	0.858
Dyslipidemia	105 (26.8)	30 (42.3)	***0.011***
Cardiovascular risk factors	240 (61.5)	57 (80.3)	***0.003***
Chronic kidney disease	12 (3.1)	16 (22.5)	**< *0.001***
Onco-hematologic	47 (12.0)	18 (25.4)	***0.005***
COPD	70 (17.9)	11 (15.5)	0.735
Transplant (SOT/SCT)	4 (1.0)	6 (8.5)	***0.001***
Neurologic	31 (7.9)	15 (21.1)	***0.002***
Rheumatologic	13 (3.3)	2 (2.8)	0.827
Liver disease	12 (3.1)	3 (4.2)	0.712
Peptic Ulcer disease	4 (1.0)	2 (2.8)	0.231
Thromboembolic disease	6 (1.5)	2 (2.8)	0.354

Thyroid disorders	18 (14.6)	2 (2.8)	0.494
Immunosuppression	31 (7.9)	10 (14.1)	0.110
Clinical symptoms, n (%)			
Cough	304 (78.1)	52 (74.3)	0.533
Fever	344 (88.9)	65 (91.5)	0.676
Dyspnea	254 (65.3)	60 (84.5)	***0.001***
Gastrointestinal	94 (24.0)	11 (15.5)	0.126
Sore throat	22 (5.6)	4 (5.6)	0.994
Anosmia/ageusia	29 (7.4)	4 (5.6)	0.803
Myalgia	86 (21.9)	8 (11.3)	***0.038***
Headache	32 (8.2)	1 (1.4)	***0.043***
Fatigue	68 (17.3)	10 (14.1)	0.606
Chest pain	23 (5.9)	3 (4.2)	0.782
Rash	2 (0.5)	0	0.546
Increased sputum production	10 (2.6)	1 (1.4)	0.561
Confusion	14 (3.7)	14 (20.3)	***< 0.001***
Days from onset of symptoms to diagnosis, Mean (SD)	8.5 (5.0)	6.7 (4.0)	***0.006***
Days from onset of symptoms to hospital admission, Mean (SD)	7.8 (4.1)	6.3 (3.8)	***0.007***
Days from onset of symptoms to therapy, Mean (SD)	7.6 (4.1)	6.2 (3.6)	***0.001***
Days from onset of symptoms to steroids, Mean (SD)	11.2 (4.9)	8.6 (3.7)	***<0.001***
Treatment, n (%)			

Hydroxychloroquine	388 (99.2)	67 (94.4)	***0.010***
Lopinavir/Ritonavir	290 (74.6)	39 (54.9)	***0.001***
Azithromycin	195 (51.2)	42 (59.2)	0.245
Interferon	187 (48.2)	27 (38.6)	0.153
Tocilizumab	165 (42.4)	24 (34.3)	0.236
Anakinra	7 (1.8)	1 (1.4)	0.822
Other treatments	69 (17.6)	16 (22.5)	0.321
PaO2/FiO2, Mean (SD)	276 (104)	193 (99)	**< *0.001***
SatO2/FiO2, Mean (SD)	294 (116)	219 (111)	**< *0.001***
Brescia-COVID19 ≥2, n (%)	62 (15.8)	31 (43.7)	**< *0.001***
ARDS, n (%)			
No	155 (39.5)	10 (14.1)	
Mild	125 (31.8)	14 (19.7)	**< *0.001***
Moderate	93 (23.7)	38 (53.5)	
Severe	19 (4.8)	9 (12.7)	
Admitted to ICU at D0	27 (6.9%)	3 (4.2%)	0.600
Laboratory outcomes, Mean (SD)			
Lymphocytes	1.077 (1,413)	796 (533)	0.133
Lactate dehydrogenase	376 (132)	459 (221)	**< *0.001***
D Dimer	1.98 (5.87)	4.89 (12.09)	***0.010***
C-reactive protein	133 (84)	164 (78)	***0.010***
Ferritin	1.092 (868)	2,563 (5.308)	**0.271**

Interleukin 6 (**IL-6**)	169 (217)	254 (212)	0.294
Chest CT (at hospital admission), n (%)			
Normal imaging	16 (4,2)	2 (2,9)	
Unilateral pneumonia	38 (9.9)	3 (4.3)	
Bilateral interstitial pneumonia	213 (55.3)	31 (44.9)	***< 0.001***
Patchy bilateral pneumonia	94 (24.4)	15 (21.9)	
Confluent bilateral pneumonia	24 (6.2)	18 (26.1)	
**Steroid treatment, n (%)**	**341 (87.0)**	**55 (77.5)**	***0.044***

Appendix Table 2. Baseline demographic and clinical characteristics of the patients according to initial steroid regimen

N=396	1 mg/kg/day Cohort (N=310)	Pulses Cohort (N=86)	p value
Gender (Men), n (%)	213 (68.7%)	63 (73.3%)	0.417
Age, Mean (SD)	65.3 (13.0)	65.8 (12.2)	0.775
Charlson score, Mean (SD)	2.03 (2.2)	2.00 (2.3)	0.916
Underlying Medical Conditions, n (%)			
High Blood pressure	148 (47.7%)	34 (39.5%)	0.177
Ischemic heart disease	63 (20.3%)	9 (10.5%)	***0.036***
Diabetes	65 (21%)	19 (22.1%)	0.821
Obesity	24 (7.7%)	5 (5.8%)	0.544
Dyslipidemia	90 (29%)	23 (26.7%)	0.678
Cardiovascular risk factors	204 (66.2%)	45 (52.3%)	***0.018***
Chronic kidney disease	19 (6.1%)	5 (5.8%)	0.914
Onco-hematologic	34 (11%)	15 (17.4%)	0.107
COPD	53 (17.1%)	18 (20.9%)	0.412
Transplant (SOT/SCT)	8 (2.6%)	1 (1.2%)	0.435
Neurologic	24 (7.7%)	11 (12.8%)	0.144
Rheumatologic	8 (2.6%)	6 (7%)	0.051
Liver disease	8 (2.6%)	2 (2.3%)	0.894
Peptic Ulcer disease	2 (0.6%)	1 (1.2%)	0.624
Thromboembolic disease	4 (1.3%)	1 (1.2%)	0.925
Thyroid disorders	11 (3.5%)	4 (4.7%)	0.636
Immunosuppression	29 (9.4%)	8 (9.3%)	0.981

Clinical symptoms at admission, n (%)			
Cough	245 (80.1%)	67 (77.9%)	0.661
Fever	281 (91.5%)	72 (85.7%)	0.111
Dyspnea	219 (71.3%)	53 (61.6%)	0.085
Gastrointestinal	75 (24.2%)	17 (19.8%)	0.390
Sore throat	18 (5.8%)	4 (4.7%)	0.679
Anosmia/ageusia	20 (6.5%)	8 (9.3%)	0.362
Myalgia	66 (21.3%)	16 (18.6%)	0.587
Headache	25 (8.1%)	4 (4.7%)	0.282
Fatigue	44 (14.2%)	19 (22.1%)	0.076
Chest pain	17 (5.5%)	6 (7%)	0.600
Rash	2 (0.6%)	0 (0%)	0.455
Increased sputum production	3 (1%)	3 (3.5%)	0.090
Confusion	10 (3.3%)	8 (9.4%)	***0.018***
Days from onset of symptoms to diagnosis, Mean (SD)	8.5 (5.4)	8.5 (4.0)	0.985
Days from onset of symptoms to hospital admission, Mean (SD)	7.5 (4.2)	8.3 (4.1)	0.118
Days from onset of symptoms to therapy, Mean (SD)	7.4 (4.2)	7.7 (3.8)	0.599
Days from onset of symptoms to steroids, mean (SD)	10.7 (5.0)	11.32 (4.0)	0.307
Number of Pulses, Mean (SD)	*3.3 (2.0)	3.5 (2.0)	0.522

Pulse dose	*		***0.015***
<250 mg	7 (10.3%)	24 (15.8%)	
250 mg	46 (67.6%)	49 (51.6%)	
> 500 mg	15 (22.1%)	11 (13.1%)	
Days from steroid 1mg/kg/d to pulses*, Mean (SD)	4.9 (3.7)	N/A	
Treatment, n (%)			
Hydroxychloroquine	307 (99.4%)	86 (100%)	0.454
Lopinavir/Ritonavir	222 (72.1%)	65 (76.5%)	0.419
Azithromycin	159 (52.8%)	49 (57.6%)	0.431
Interferon	146 (47.9%)	40 (46.5%)	0.903
-before or together with steroids	138 (93.9%)	33 (94.3%)	0.999
-after steroids	9 (6.1%)	2 (5.7%)	
Tocilizumab	130 (42.2%)	47 (54.7%)	0.05
-before or together with steroids	77 (59.7%)	25 (59.5%)	0.999
-after steroids	52 (40.3%	17 (40.5%)	
Anakinra	4 (1.3%)	4 (4.7%)	0.071
-before or together with steroids	2 (66.7%)	0	0.4
-after steroids	1 (33.3%)	3 (75%)	
Other treatments**			
PaO2/FiO2, Mean (SD)	261.4 (110.4)	270.5 (118.4)	0.544
SatO2/FiO2, Mean (SD)	286.7 (123.6)	300.6 (125.6)	0.543
Brescia-COVID19 ≥2, n (%)	62 (20%)	15 (17.4%)	0.596

ARDS, n (%)			0.110
No	115 (37.1%)	40 (46.5%)	
Mild	82 (26.5%)	14 (16.3%)	
Moderate/ Severe	113 (36.5%)	32 (37.2%)	
Admitted to ICU at D0	28 (9%)	2 (2.3%)	0.038
Laboratory (day 0), Mean (SD)			
Lymphocyte counts	1040.6 (1522 (1)	879.2 (412.0)	0.384
Lactate dehydrogenase	396.7 (157.7)	395.6 (141.5)	0.963
D- Dimer	2.1 (6.4)	3.6 (10.6)	0.295
C-reactive protein	139.6 (84.0)	145.7 (88.4)	0.611
Ferritin	1486.8 (2589.2)	1042.7 (783.6)	0.340
Interleukin 6 (**IL-6**)	195.1 (230.3)	201.1 (229.7)	0.921
Chest CT (at hospital admission), n (%)			0.275
Normal imaging	14 (4.6%)	2 (2.4%)	
Unilateral pneumonia	24 (7.9%)	5 (5.9%)	
Bilateral interstitial pneumonia	169 (55.6%)	48 (56.5%)	
Patchy bilateral pneumonia	75 (24.7%)	18 (21.2%)	
Confluent bilateral pneumonia	22 (7.2%)	12 (14.1%)	

*In 70 (22.6%) patients that received steroid pulses after a 1 mg/kg/day regimen **Including DRVr/ doxycycline/ clarithromycin and other antibiotics

COPD: chronic obstructive pulmonary disease; SOT: solid organ transplantation; SCT: stem cell transplantation; D0: day 0; SD: standard deviation; N/A: non-applicable; PaO2/FiO2: arterial oxygen tension/inspiratory oxygen fraction; SatO2/FiO2: oxygen saturation /inspiratory oxygen

fraction; ICU: intensive care unit; ARDS: acute respiratory distress syndrome; Brescia-COVID19: respiratory severity scale Brescia-COVID-19; CT: computed tomography scan; IL-6: Interleukin 6; DRVr: ritonavir-boosted darunavir

Appendix Table 3. Baseline demographic and clinical characteristics of the patients initially receiving 1 mg/kg/day according to subsequent need for steroid pulses

N=310	1 mg/kg/day followed by steroid pulses (N=70)	1 mg/kg/d (N=240)	p value
Gender (Men), n (%)	50 (71.4%)	163 (67.9%)	0.577
Age, Mean (SD)	67.2 (12.7)	64.8 (13.1)	0.169
Charlson score, Mean (SD)	2.4 (2.5)	1.9 (2.2)	0.102
Underlying medical conditions, n (%)	58(82.8%)	180 (75%)	0.171
High Blood pressure	34 (48.5%)	114 (47.5%)	0.875
Ischemic heart disease	12 (17.1%)	51 (21.2%)	0.452
Diabetes	21 (30%)	44 (18.3%)	***0.035***
Obesity	6 (8.5%)	18 (7.5%)	0.768
Dyslipidemia	23 (32.8%)	67 (27.9%)	0.423
Cardiovascular risk factors	(68.11%)	157 (65.6%)	0.707
Chronic kidney disease	5 (7.1%)	14 (5.8%)	0.688
Onco-hematologic	10 (14.2%)	24 (10%)	0.313
COPD	8 (11.4%)	45 (18.7%)	0.152
Transplant (SOT/SCT)	2 (2.8%)	6 (2.5%)	0.868
Neurologic	12 (17.1%)	12 (5%)	***0.001***
Rheumatologic	3 (4.3%)	5 (2.1%)	0.386
Liver disease	2 (2.9%)	6 (2.5%)	0.999

Peptic Ulcer disease	0	2 (0.8%)	0.999
Thromboembolic disease	2 (2.9%)	2 (0.8%)	0.221
Thyroid disorders	2 (2.9%)	9 (3.8%)	0.999
Immunosuppression	11 (15.9%)	18 (7.5%)	***0.034***
Clinical symptoms at admission, n (%)			
Cough	55 (80.9%)	190 (79.8%)	0.999
Fever	64 (92.7)	217 (91.2%)	0.679
Dyspnea	54 (79.4%)	165 (69%)	0.095
Gastrointestinal	15 (21.4%)	60 (25%)	0.635
Sore throat	5 (7.1%)	13 (5.4%)	0.567
Anosmia/ageusia	6 (8.6%)	14 (5.8%)	0.412
Myalgia	11 (15.7%)	55 (22.9%)	0.246
Headache	3 (4.3%)	22 (9.2%)	0.222
Fatigue	10 (14.3%)	34 (14.2%)	0.999
Chest pain	6 (8.6%)	11 (4.6%)	0.231
Rash	0	2 (0.8%)	0.999
Increased sputum production	1 (1.4%)	2 (0.8%)	0.537
Confusion	4 (6.1%)	6 (2.5%)	0.234
Days from onset of symptoms t to diagnosis, Mean (SD) (Range)	8.5 (4.9)	8.5 (5.5)	0.972

Days from onset of symptoms to hospital admission, Mean (SD)	7.5 (4.3)	7.5 (4.2)	0.940
Days from onset of symptoms to therapy, Mean (SD)	7.3 (4.2)	7.4 (4.2)	0.869
Days from onset of symptoms to steroids, Mean (SD)	9.3 (4.4)	11.1 (5.1)	***0.010***
Days from steroid 1mg/kg/day to pulses* mean (SD)	4.9 (3.7)	N/A	
Treatment, n (%)			
Hydroxychloroquine	69 (100%)	238 (99.2%)	0.999
Lopinavir/Ritonavir	48 (70.6%)	174 (72.5%)	0.761
Azithromycin	41 (61.2%)	118 (50.4%)	0.129
Interferon	40 (57.9%)	106 (44.9%)	0.056
-before or together with steroids	34 (82.9%)	104 (98.1%)	***0.002***
-after steroids	7 (17.1%)	2 (1.9%)	
Tocilizumab	48 (68.5%)	82 (34.4%)	***0.000***
-before or together with steroids	18 (37.5%)	59 (72.8%)	***0.000***
-after steroids	30 (62.5%)	22 (27.2%)	
Anakinra	2	1	0.999
-before or together with steroids	1 (50%)	1 (100%)	
-after steroids	1 (50%)	0	

-after steroids			
Other treatments**	13 (18.6%)	33 (13.8%)	0.341
PaO2/FiO2, Mean (SD)	233.4 (111.5)	271.6 (108.5)	***0.017***
SatO2/FiO2, Mean (SD)	250.8 (135.7)	298.2 (117.9)	0.055
Brescia-COVID19 ≥2, n (%)	18 (25.7%)	44 (18.3%)	0.174
ARDS, n (%)			***0.017***
No	16 (22.9%)	99 (41.3%)	
Mild	21 (30%)	61 (25.4%)	
Moderate/ Severe	33 (47.1%)	80 (33.3%)	
Admitted to ICU at D0	5 (7.1%)	23 (9.6%)	0.641
Laboratory (D0), Mean (SD)			
Lymphocyte counts	1247.8 (2492.6)	970.4 (1002.0)	0.404
Lactate dehydrogenase	437.4 (157.9)	382.7(155.6)	0.021
D- Dimer	2.0 (3.6)	2.2 (7.1)	0.902
C-reactive protein	165.4 (79.3)	130.5 (84.0)	0..05
Ferritin	1070 (838.4)	1633 (2973.4)	0.369
Interleukin 6 (**IL-6**)	232.2 (246.6)	181.2 (225.5)	0.470
Chest CT (at hospital admission), n (%)			***0.029***
Normal imaging	3 (4.4%)	11 (4.7%)	
Unilateral pneumonia	4 (5.9%)	20 (8.5%)	

Bilateral interstitial pneumonia	36 (52.9%)	133 (56.4%)
Patchy bilateral pneumonia	14 (20.6%)	61 (25.8%)
Confluent bilateral pneumonia	11 (16.2%)	11 (4.7%)

*In 70 (22.6%) patients that received steroid pulses after a 1 mg/kg/day regimen

**Including DRVr/ doxycycline/ clarithromycin and other antibiotics.

COPD: chronic obstructive pulmonary disease; SOT: solid organ transplantation; SCT: stem cell transplantation; ICU: intensive care unit; D0: day 0; SD: standard deviation; N/A: non-applicable; PaO2/FiO2: arterial oxygen tension/inspiratory oxygen fraction; SatO2/FiO2: oxygen saturation /inspiratory oxygen fraction; ARDS: acute respiratory distress syndrome; Brescia-COVID19: respiratory severity scale Brescia-COVID-19; CT: computed tomography scan; IL-6: Interleukin 6; DRVr: ritonavir-boosted darunavir

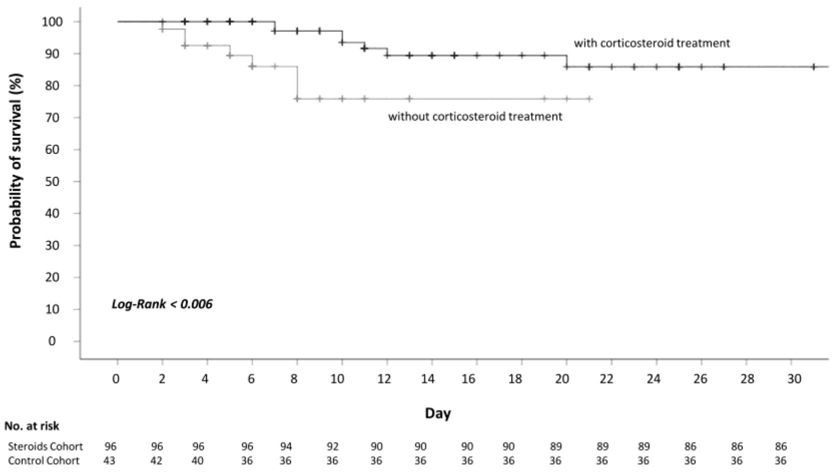

Appendix Figure 1. Probability of survival from D0 to hospital discharge of patients with SARS-COV-2 infection, according steroid exposure (Mild ARDS).

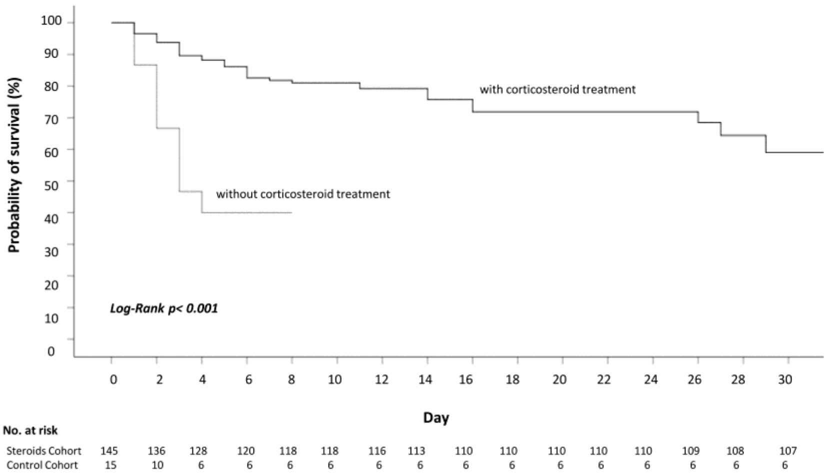

Appendix Figure 2. Probability of survival from D0 to hospital discharge of patients with SARS-COV-2 infection, according to steroid exposure (Moderate-severe ARDS).
